# Reference-free deconvolution of complex samples based on cross-cell-type differential analysis: Systematic evaluations with various feature selection options

**DOI:** 10.3389/fgene.2025.1570781

**Published:** 2025-05-30

**Authors:** Weiwei Zhang, Zhonghe Tian, Ling Peng

**Affiliations:** School of Mathematics Information, Shaoxing University, Shaoxing, China

**Keywords:** reference-free deconvolution, feature selection, cross-cell-type differential analysis, cell compositions, gene expression, DNA methylation

## Abstract

**Introduction:**

Genomic and epigenomic data from complex samples reflect the average level of multiple cell types. However, differences in cell compositions can introduce bias into many relevant analyses. Consequently, the accurate estimation of cell compositions has been regarded as an important initial step in the analysis of complex samples. A large number of computational methods have been developed for estimating cell compositions; however, their applications are limited due to the absence of reference or prior information. As a result, reference-free deconvolution has the potential to be widely applied due to its flexibility. A previous study emphasized the importance of feature selection for improving estimation accuracy in reference-free deconvolution.

**Methods:**

In this paper, we systematically evaluated five feature selection options and developed an optimal feature-selection-based reference-free deconvolution method. Our proposal iteratively searches for cell-type-specific (CTS) features by integrating cross-cell-type differential analysis between one cell type and the other cell types, as well as between two cell types and the other cell types, and performs composition estimation.

**Results and discussion:**

Comprehensive simulation studies and analyses of seven real datasets show the excellent performance of the proposed method. The proposed method, that is, reference-free deconvolution based on cross-cell-type differential (RFdecd), is implemented as an R package at https://github.com/wwzhang-study/RFdecd.

## 1 Introduction

Genomic and epigenomic data obtained from complex samples represent a weighted average of signals originating from multiple cell types, rather than individual measures for each feature across different cell types present in the mixture (Houseman et al., 2012; Jaffe and Irizarry, 2014; Shen-Orr et al., 2010; Zhong and Liu, 2011). For instance, DNA methylation profiles derived from whole blood reflect contributions from heterogeneous cell populations, such as lymphocytes (e.g., T cells and B cells), granulocytes (e.g., neutrophils), and monocytes (Zhang et al., 2021; Zheng et al., 2018). Similarly, tumor samples are composed of heterogeneous cellular mixtures, including malignant cells, stromal cells (e.g., fibroblasts), vascular endothelial cells, and immune cell subsets (e.g., T cells and macrophages), which collectively constitute the tumor microenvironment (Marusyk et al., 2012; Yadav and De, 2015). Consequently, differences in cell compositions can confound many relevant analyses, including differential analysis (Zhang et al., 2020; Zheng X. et al., 2017), and cell-type classification (Chen et al., 2024). Moreover, cell compositions serve as a crucial foundation for forecasting disease progression and patient prognosis (Dou et al., 2024; Ribas and Wolchok, 2018). Therefore, the accurate estimation of cell compositions from high-throughput data of complex samples is of great significance.

Cell composition analysis can be assessed through both *in vitro* experimental and *in silico* computational approaches. Many experimental approaches, such as fluorescence-activated cell sorting (FACS) and immunohistochemistry (IHC), along with advanced techniques like single-cell transcriptome analysis and multi-omics sequencing, provide cellular composition information (Sturm et al., 2019). However, these *in vitro* methods are either limited by their processing capacity or remain too expensive and labor-intensive for large-scale clinical use. To address this issue, *in silico* computational techniques known as “deconvolution” have been devised as alternatives. These approaches can generally be divided into two main categories: reference-based (RB) methods (Clarke et al., 2010; Gong et al., 2011; Hattab et al., 2017; Newman et al., 2015; Teschendorff et al., 2017) and reference-free (RF) methods (Brunet et al., 2004; Houseman et al., 2016; Kang et al., 2019; Rahmani et al., 2018). RB methods require the use of reference panel data that can be derived from purified tissues or annotated single-cell experiments. The proportion of each cell type is then determined using techniques such as constrained linear regression or support vector regression (Newman et al., 2015). It has been reported that RB methods generally provide more accurate and robust estimations than RF methods (Newman et al., 2015; Teschendorff et al., 2017; Zheng S. C. et al., 2017). Nevertheless, RB methods often encounter limitations due to the accessibility of appropriately matched reference panels that adequately match the target population in terms of key biological characteristics. Currently, the available reference data predominantly pertain to only a handful of extensively researched tissue types, such as the blood, breast, and brain, sourced from a relatively small number of individuals. Additionally, in scenarios where substantial disparities exist in clinical conditions and phenotypes between the intricate samples under study and the reference data, RB methods may lead to imprecise estimations of proportions (Li and Wu, 2019). For tissues without proper references, RF methods provide a better solution (Ferro Dos Santos et al., 2024; Rahmani et al., 2017). RF deconvolution is a computational framework that estimates cellular heterogeneity without requiring prior cell-type marker information by simultaneously inferring cell-type-specific (CTS) signatures and proportions directly from bulk data. Classical approaches including non-negative matrix factorization (NMF) (Brunet et al., 2004) and https://MeDeCom (Lutsik et al., 2017) require biological priors to resolve rotational ambiguity. Recent advancements diversify RF strategies: hierarchical latent variable models [e.g., MMAD (Houseman et al., 2016)] enhance biological interpretability through structured latent variables, whereas Bayesian frameworks like BayesPrism (Chu et al., 2022) improve identifiability *via* prior integration. Feature selection strategies, such as co-functional grouping (Deng et al., 2023), optimize biological relevance at computational cost. Spatial extensions like STANDS (Xu et al., 2024) enable reference-free tissue analysis *via* graph neural networks, contingent on specialized spatial inputs. Despite methodological advancements, RF frameworks continue to face inherent parameter estimation challenges in simultaneously estimating high-dimensional parameters for cellular signatures and proportions, often leading to reduced estimation accuracy. Therefore, it is worthwhile to investigate potential strategies that can improve RF deconvolution.

In this paper, we systematically evaluated five feature-selection options in reference-free deconvolution and developed an optimal feature-selection-based reference-free deconvolution based on cross-cell-type differential (RFdecd) analysis using an iteration algorithm to search for cell-type-specific features and perform cell composition estimation. We evaluate the proposed method through extensive simulation studies and analysis of seven real data. Our proposed method is implemented in the latest version of the RFdecd package, which is freely available at https://github.com/wwzhang-study/RFdecd.

## 2 Materials and methods

### 2.1 Data model

RF deconvolution uses a raw data matrix Y from complex samples to estimate cell-type profiles and cell compositions. In mathematical terms, this problem can be formulated as shown in Equation 1:

Y=WH+ϵ, (1)

where W is an m×K cell-type profile matrix for m cell-type-specific features in K cell types; H is a K×n cell-type-specific mixing proportion matrix (rows = K cell types and column = n samples with proportions summing to 1) for K cell types in n samples, and the entries of H are required to be non-negative, and every column sums up to one; ϵ is an m×n error matrix. The goal of this study was to use Y to estimate H .

**FIGURE 1:**
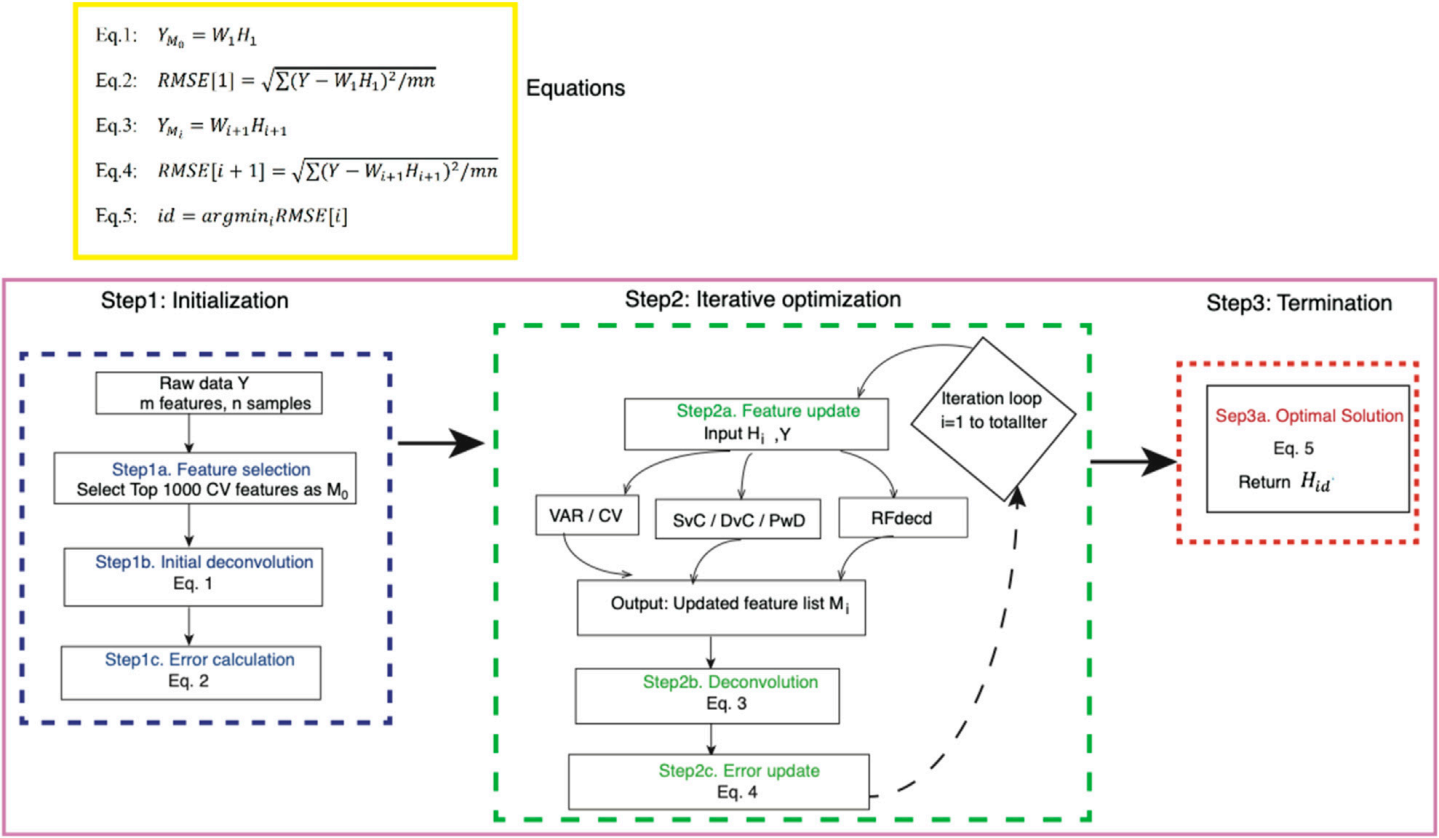
Workflow of the proposed method. Our algorithm starts with raw data Y and consists of three main steps. In step 1 (initialization), the top 1,000 features ( $M_{0}$ ) with the largest coefficient of variation (CV) are selected from Y . RF deconvolution is performed on the reduced matrix $Y_{M_{0}}$ to estimate the initial cell-type proportion matrix $H_{1}$ and compute the reconstruction error (RMSE [1]). In step 2 (iterative optimization), starting from i=1 , the algorithm iterates for totalIter cycles. At each iteration i , the current proportion matrix $H_{i}$ and raw data Y are used to update the feature list $M_{i}$ through six feature-selection options (VAR, CV, SvC, DvC, PwD, and RFdecd). RF deconvolution on the updated matrix $Y_{M_{i}}$ generates the proportion matrix $H_{i+1}$ and updates the error RMSE[i+1]. In step 3 (termination), the optimal proportion matrix $H_{id}$ is selected as the iteration with the minimal RMSE.

Figure 1 shows the workflow of the proposed algorithm, which consists of three main phases. The initialization phase begins by selecting the top 1,000 features ( $M_{0}$ ) with the highest coefficient of variation (CV) from the raw data matrix Y , generating a reduced matrix $Y_{M_{0}}$ that undergoes RF deconvolution to estimate initial cell-type profile matrix $W_{1}$ and proportion matrix $H_{1}$ , with the reconstruction error RMSE[1] calculated as the root mean squared error between the reconstructed observation $\hat{Y}=W_{1}H_{1}$ and the original observation Y . The iterative optimization phase then cyclically updates the feature list $M_{i}$ using six feature-selection options, namely, variance (VAR), CV, single-vs-composite (SvC), dual-vs-composite (DvC), pairwise-direct (PwD), and RFdecd, at each iteration i (1 ≤i≤totalIter ), followed by the re-estimation of $W_{i+1}$ and $H_{i+1}$ through RF deconvolution on $Y_{M_{i}}$ and recalculation of RMSE[i+1]. After completing all iterations, the termination phase identifies and returns the optimal proportion matrix $H_{id}$ corresponding to the iteration with minimal root mean squared error (RMSE). A formal pseudocode of the complete algorithm is provided in Supplementary Algorithm 1.

The algorithm uses six feature-selection options during iterative optimization. Initial approaches include VAR and CV, which select the top 1,000 features based on VAR or CV in the estimated cell-type profiles. Building on the previous work of Li and Wu (2019), who demonstrated that cross-cell-type differential analysis enhances feature selection and subsequent proportion estimation, we further developed three strategies: SvC, reflecting the comparison between one target cell type and a composite group of all other cell type; DvC, indicating the joint analysis of two specified cell types against the remaining composite population; and PwD, emphasizing direct feature selection between two explicitly contrasted cell types without composite interference. For illustration, a sample is considered with four cell types ( K=4 ). SvC performs differential analysis between each cell type $k\left(k=1,2,3,4\right)$ and the composite of the remaining three, that is, comparing cell type 1 with cell types 2, 3, and 4; cell type 2 with cell types 1, 3, and 4; cell type 3 with cell types 1, 2, and 4; and cell type 4 with cell types 1, 2, and 3. For each comparison, the top $\left\lceil \frac{1000}{K}*1.2\right\rceil$ features are selected from sorted p-values, where ⌈·⌉ denotes the ceiling operation. Because different cell types may have overlapped cross-cell-type features, we choose 1.2 times the desired value. We merged the feature list across cell types, removed duplicate features, and ultimately obtained the desired feature list. DvC compares the pairs of cell types (e.g., k+l ) against the remaining two, selecting top $\left\lceil \frac{1000}{K}*1.2\right\rceil$ features per comparison. PwD directly contrasts individual cell-type pairs (e.g., k vs. l ) using the same feature-selection threshold. Empirical evaluations revealed that cross-cell-type differential analysis-based strategies (SvC, DvC, and PwD) outperformed variance-based methods (VAR/CV), with SvC and DvC achieving superior accuracy over PwD. To optimize performance, we designed RFdecd, a hybrid approach integrating SvC and DvC. Specifically, we first conducted SvC and obtained cell-type-specific features for each cell type. For each cell type, we selected the top 100 cell-type-specific features following a systematic allocation strategy (100 features × 4 cell types = 400) to achieve balanced representation and ensured that the selected feature overlap of any two cell types was empty. Finally, a total of 400 features were obtained for these four cell types under the feature-selection option SvC. Then, we conducted DvC and obtained cell-type-specific features for each comparison. Due to the same results between cell types 1 and 2 versus 3 and 4, as well as between cell types 3 and 4 versus 1 and 2, we obtained only three comparisons in the end. For each comparison, we selected the top 100 features using equivalent allocation (100 features × 3 comparisons = 300) that were not included in the previous 400 features and ensured that the selected features did not overlap with each other. Thus, we have obtained a total of 700 cell-type-specific features. Next, we selected the top 300 features from the sorted p-values of SvC and DvC and ensured that the intersection of the top 300 features with the previous 700 features is an empty set. These 1,000 identified features replace $M_{0}$ and are then used in a new iteration. The algorithm iterates for a number of times and then stops. Based on our experience, 30 iterations are sufficient for gene expression and DNA methylation datasets with four cell types and 100 samples, achieving strong correlations (>0.95) between estimated and true proportions (Supplementary Figure S1). More iterations are required for studies with smaller sample sizes (e.g., less than 50) or more cell types (e.g., six or more). In our software, the users can specify the total number of iterations. In each iteration, the six algorithms would calculate the RMSE between the reconstructed observation $\hat{Y}$ and true observation Y , and the estimated proportion matrix corresponding to the iteration with the smallest RMSE would be chosen as the final estimation. Our algorithm is not limited by specific deconvolution methods; therefore, most of the existing RF methods can be used in combination with this procedure. In this study, we used the RF algorithm deconf (Repsilber et al., 2010) for gene expression microarray data and RefFreeEWAS (Houseman et al., 2016) for DNA methylation microarray data.

### 2.2 Selection of features using cross-cell-type differential analysis

We denote all the observed data for the p -th feature as $Y_{p}={\left[Y_{p1},Y_{p2},\ldots ,Y_{pn}\right]}^{T}$ . It is assumed that the cell-type proportions of all samples are known, and the proportion of sample s is denoted as $\theta_{s}=\left(\theta_{s1},\theta_{s2},\ldots ,\theta_{sK}\right)$ . Consequently, the observed data can be characterized using a linear model: $E\left[Y_{p}\right]=W\beta_{p}$ , where

$$W=\left[\begin{array}{c} \theta_{11}\\ \theta_{21}\\ \vdots \\ \theta_{n1} \end{array}\;\begin{array}{c} \theta_{12}\\ \theta_{22}\\ \vdots \\ \theta_{n2} \end{array}\;\begin{array}{c} \ldots \\ \ldots \\ \vdots \\ \ldots \end{array}\;\begin{array}{c} \theta_{1K}\\ \theta_{2K}\\ \vdots \\ \theta_{nK} \end{array}\right],\beta =\left[\mu_{p1},\mu_{p2},\ldots ,\mu_{pK}\right].$$ (2)

In Equation 2, $\mu_{pk}$ denotes the average level of the p -th feature in the k -th cell type. It has been proven that the parameterization above allows great flexibility in hypothesis testing (Li et al., 2019). Here, we highlight the null ( $H_{0}$ ) and alternative ( $H_{1}$ ) hypotheses for SvC, DvC, and PwD:

(1) SvC: testing the difference in the p -th feature between cell type k and the other cell types as (Equation 3)
$$H_{0}:\mu_{\text{pk}}‐\frac{1}{K‐1}\sum_{i\neq k}\mu_{\text{pi}}=0\text{ vs}.\;H_{1}:\mu_{\text{pk}}‐\frac{1}{K‐1}\sum_{i\neq k}\mu_{\text{pi}}\neq 0.$$ (3)
(2) DvC: testing the difference in the p -th feature between cell types k,l and the other cell types as (Equation 4)
$$H_{0}:\left(\mu_{pk}+\mu_{pl}\right)-\sum_{i\neq k,l}\mu_{pi}=0\;vs.\;H_{1}:\left(\mu_{pk}+\mu_{pl}\right)-\sum_{i\neq k,l}\mu_{pi}\neq 0.$$ (4)
  This formulation assesses whether the combined effect of groups k and l is statistically equivalent to the aggregated effect of all other groups, moving beyond simplistic pairwise “mean of means” comparisons to avoid oversimplification while accounting for potential synergistic interactions.
(3) PwD: testing the difference in the p -th feature between cell type k and cell type l as (Equation 5)

$$H_{0}:\mu_{pk}-\mu_{pl}=0\text{ vs}.\;H_{1}:\mu_{pk}-\mu_{pl}\neq 0.$$ (5)

The ordinary least squares (OLS) method can be used to fit the linear model described above, and the corresponding test statistics and p-values can be derived from this estimation.

### 2.3 Datasets

#### 2.3.1 Simulation datasets

We designed two simulation studies based on real datasets: one for the gene expression data and the other for the DNA methylation data. The simulated data ( Y ) were based on two randomly generated matrices: a cell-type-specific reference matrix ( W ) and a cell-type-specific proportion matrix ( H ). Proportion matrix H of the two simulation studies was simulated from a Dirichlet distribution with parameters (0.968, 4.706, 0.496, and 0.347) for four cell-type settings and (0.89, 4.12, 0.47, 0.33, 0.61, and 1.02) for six cell-type settings. However, the generation processes of reference matrix W in the two simulation studies were different. For gene expression data, W was generated based on the immune dataset from the Gene Expression Omnibus (GEO) with accession number GSE11058 (Abbas et al., 2009). This dataset contains gene expression profiles from four types of immune cells (Jurkat, LM-9, Raji, and THP-1), and each has measurements from three replicated samples. We calculated the mean and variance from the log-expression values across the three replicated samples and simulated them using a log-normal distribution with estimated means and variances. For DNA methylation data, we simulated W based on DNA methylation 450K array data of purified human blood cells from GEO (accession number GSE35069) (Reinius et al., 2012). This dataset contains the DNA methylation profiles from six types of blood cells, namely, CD4 T, CD8 T, CD56 natural killer (CD56NK), B cell, monocyte (Mono), and granulocyte (Gran), and each cell type has measurements from six replicated samples (Reinius et al., 2012). In a simulation study that assumed complex samples consisting of four cell types, we combined CD4 T, CD8 T, and CD56NK to one pseudo-cell-type when estimating the cell-type-specific mean and variance of each feature. In this setting, W is randomly generated from the beta distributions using the estimated parameters. Eventually, matrix Y was simulated by multiplying these two matrices and adding small Gaussian noises. For all simulation settings, the results from 100 Monte Carlo experiments were summarized and presented.

#### 2.3.2 Real datasets

In the real data analysis, a total of seven datasets were retrieved from the Gene Expression Omnibus (GEO) database, accessible at https://www.ncbi.nlm.nih.gov/geo/ under the following accession IDs: Mouse-Mix data by Shen-Orr et al. (2010) with accession ID GSE19830, Immune data by Abbas et al. (2009) with accession ID GSE11058, Aging data by Hannum et al. (2013) with accession ID GSE40279, Rheumatoid Arthritis data by Liu et al. (2013) with accession ID GSE42861, European Prospective Investigation into Cancer and Nutrition data by Riboli et al. (2002) with accession ID GSE51032, and two Schizophrenia datasets by Hannon et al. (2016) with accession IDs GSE80417 and GSE84727, abbreviated as Hannon et al. I and Hannon et al. II, respectively. We normalized DNA methylation data using quantile normalization. In the simulation and seven datasets, we calculated the mean Pearson correlation coefficient (mean PCC) and mean absolute error (MAE) between the estimated and true proportions across the four cell types to evaluate the performance of our proposed algorithm. A higher mean PCC and lower MAE are expected for a better method.

## 3 Results

### 3.1 Simulation

**FIGURE 2:**
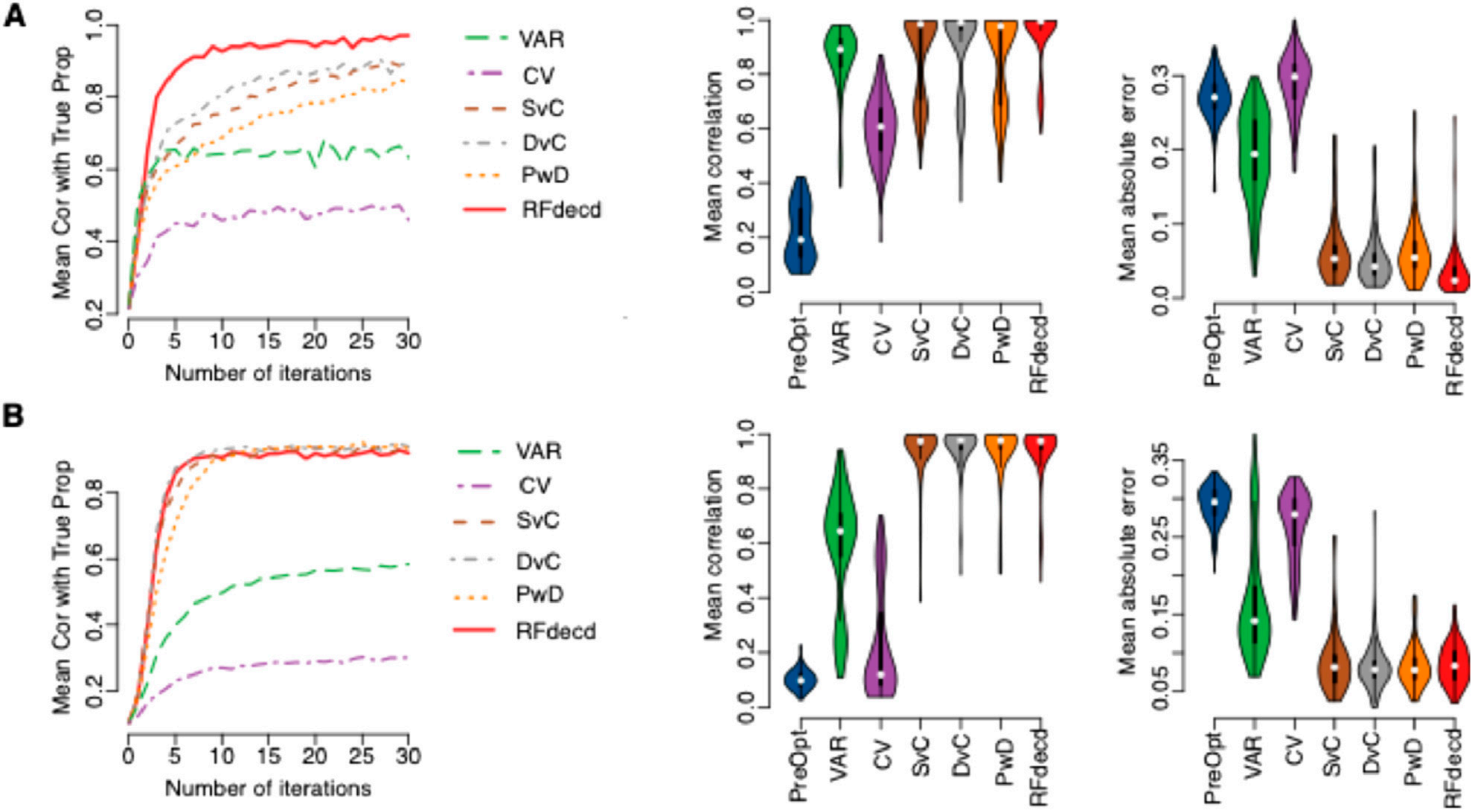
Performance of our algorithm on synthetic mixtures. **(A)** Gene expression dataset (GSE19830); **(B)** DNA methylation dataset (GSE35069). Left panels: boxplots of mean Pearson correlations between estimated and true cell-type proportions across four cell types for six methods (VAR, CV, SvC, DvC, PwD, and RFdecd) over 30 iterations. Middle and right panels: boxplots comparing mean Pearson correlations (middle) and mean absolute errors (right) for seven methods, including the baseline “PreOpt” (initial deconvolution using the top 1,000 CV-selected features without iteration). Results for the six iterative methods reflect the lowest RMSE across 30 iterations. All metrics are aggregated from 100 Monte Carlo simulations with 100 samples per simulation.

First, we evaluated the performance of our method in simulation based on gene expression and DNA methylation datasets with four cell types. A total of 100 samples were generated using the simulation steps described in Section 2.4. Figure 2A is based on gene expression, and Figure 2B is based on DNA methylation. The left panel of Figure 2 shows the mean Pearson correlations between the estimated and true proportions across the four cell types at the initial point (number of iterations = 0) and after 30 iterations of application of the six proposed methods. The middle and right panels of Figure 2 show the mean Pearson correlation and mean absolute error, respectively, between the estimated and true proportions across the four cell types using the seven methods. Here, the estimated proportions of PreOpt refer to the initial cell-type proportion estimates prior to the first iteration of optimization (equivalent to iteration count = 0), derived directly from raw input features without feature selection or model refinement, and the estimated proportions of the other six methods are based on the results with the smallest RMSE in 30 iterations. The left panel clearly shows that the mean correlations between the estimated and true proportions continue to increase during the iterations for all the six methods. The improvements are dramatic for all six methods, particularly for the four methods: RFdecd, SvC, DvC, and PwD, which are based on cross-cell-type differential analysis. For the gene expression data, the mean correlation at the number of iterations = 0 was 0.217. However, at seven iterations, the mean correlation of VAR was 0.443, CV was 0.659, SvC was 0.724, DvC was 0.751, PwD was 0.637, and RFdecd was 0.911. From the middle and right panels, whether for gene expression data or DNA methylation data, compared with “PreOpt” (number of iterations = 0), the mean correlation across the four cell types significantly increased, and the mean absolute error significantly decreased. RFdecd achieved the best performance, followed by DvC and SvC. Similar results were obtained for DNA methylation data (lower panel of Figure 2). It is worth mentioning that the improvement in DNA methylation was not as rapid as that in gene expression (the mean Pearson correlation was 0.911 for seven iterations of gene expression and 0.907 for 10 iterations of DNA methylation). This computational challenge was reflected in our benchmarking tests: on a standard laptop (Apple M1 8-core processor and 16 GB unified memory), the algorithm analyzed 54,675 genes across 100 samples in 7.8 min per run while processing 459,226 CpG sites with equivalent sample size required 23.8 min. The increased computational demand for methylation data further supports the notion that its higher feature complexity impacts optimization efficiency. Thus, we suggest more iterations of the DNA methylation data with smaller sample sizes.

**FIGURE 3:**
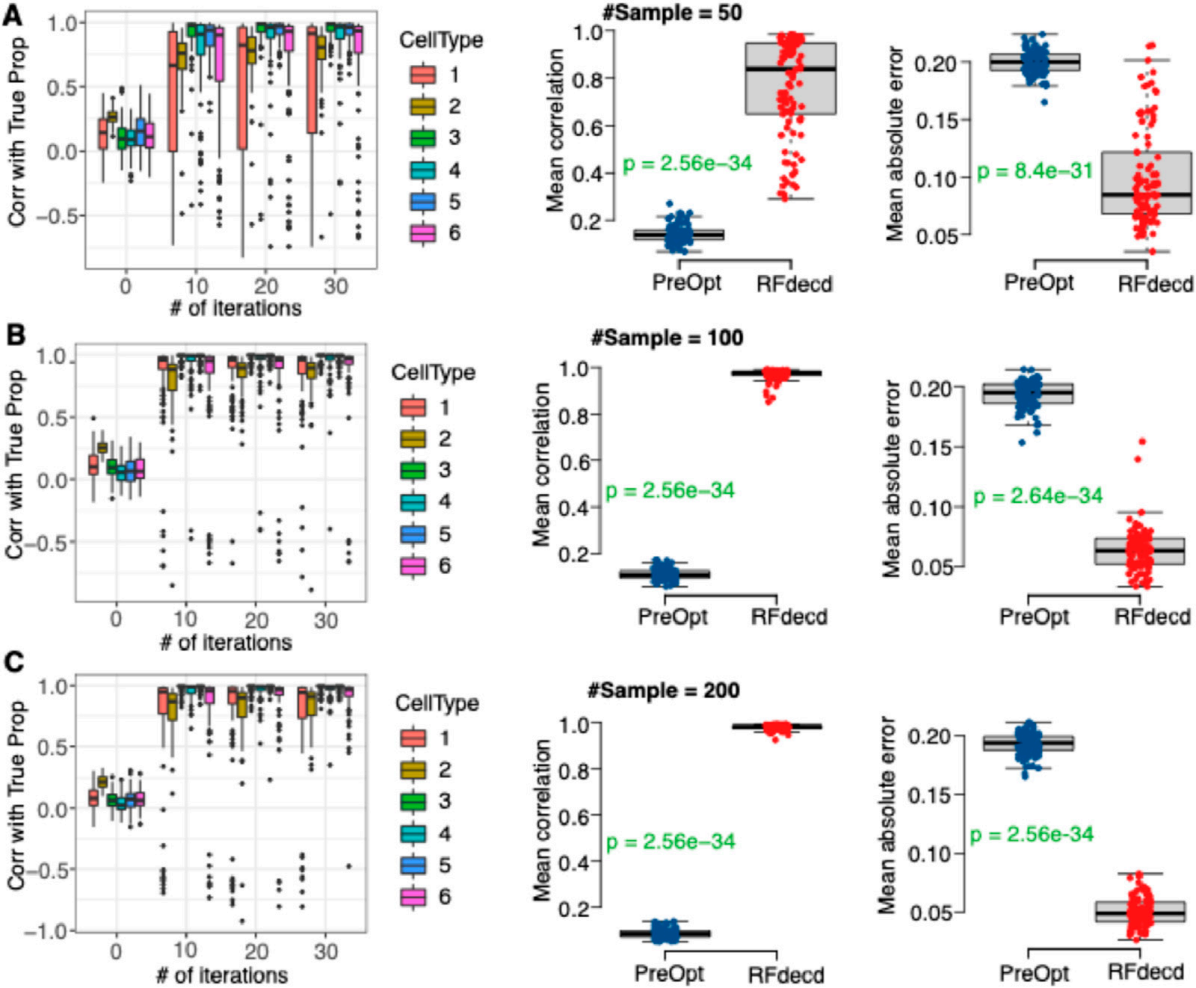
The Performance of RFdecd on synthetic mixtures based on DNA methylation dataset with six cell types under sample sizes of 50, 100 and 200 (GSE35069). **(A)** Top row (sample size 50). Left panel: boxplots of Pearson correlations between the estimated and true proportions by the number of iterations for each of the six cell types. Middle and right panels: boxplots of mean Pearson correlations and mean absolute errors between estimated and true proportions over six cell types from “PreOpt” and RFdecd. **(B)** Middle row (sample size 100): Panels for 100 samples. **(C)** Bottom row (sample size 200): Panels for 200 samples. The estimated proportions of “PreOpt” are obtained using the top 1000 features by CV for observed data as inputs for RF deconvolution (i.e., number of iterations = 0). The estimated proportions of RFdecd are based on the results with the smallest RMSE over 30 iterations. From the top panel to bottom panel, sample size increases from 50, 100 to 200. P-values for each panel were obtained using the rank-sum test. The presented results are summarized over 100 Monte Carlo simulation experiments.

Next, we examined the effect of the number of cell types in the mixture for different sample sizes. We use DNA methylation data (GSE35069) to generate simulation data with six cell types (CD4 T, CD8 T, CD56NK, B cell, Mono, and Gran). The simulation details are presented in Section 2.4. As shown in Figure 3, RFdecd consistently achieves higher correlations and lower mean absolute errors than “PreOpt.” We also observed that RFdecd had increased correlations and decreased errors when the sample size increased from 50 to 200 (the mean Pearson correlation is 0.77 for a sample size of 50 compared to 0.98 for a sample size of 200, and the mean absolute error is 0.10 for a sample size of 50 compared to 0.05 for a sample size of 200). Furthermore, compared to Figure 2B, under the same number of iterations, the proportion estimations for four cell types are more accurate than that for six cell types (the mean Pearson correlation over 10 iterations is 0.927 for four cell types, whereas it is only 0.88 for six cell types). These findings suggest that in scenarios with a limited sample size (e.g., ≤ 50), we should consider combining similar cell types to define a smaller number of cell types ( ≤4 ) and utilize the RFdecd method. The experimental design should necessitate the analysis of a greater number of cell types, augmenting the sample size emerges as the most efficacious strategy to enhance the precision of deconvolution.

To further investigate how cell-type proportion influence the deconvolution accuracy, we designed a dual-pronged validation strategy targeting both the generation and perturbation of the proportion matrix H . First, using the gene expression dataset (GSE19830), we generated the H matrix under three Dirichlet parameter configurations: (1) a uniform distribution (1, 1, 1, 1) modeling balanced cell-type proportions, (2) a moderately skewed distribution (2, 3, 0.5, 0.5) reflecting intermediate variability in cell-type abundance, and (3) an extreme distribution (5, 5, 0.01, 0.01) including near-zero proportions for two cell types. The analysis of 100 synthetic samples per configuration across eight methods (Supplementary Figure S2) revealed that RFdecd consistently outperformed PreOpt, VAR, CV, SvC, DvC, and PwD, achieving comparable accuracy to the CTS method—a gold-standard approach using 1,000 real CTS markers for direct proportion estimation.

Notably, under the extreme configuration, where two cell types approached sparsity ( $\alpha =\left[5,5,0.01,0.01\right]$ ), all methods exhibited diminished accuracy for rare populations, yet RFdecd maintained superior robustness. Second, to assess dependency on initial conditions, we permuted the initial matrix H (“RFdecd-perm”) and applied iterative optimization. Results demonstrated consistently high correlations and low MAE (mean PCC is 0.951 and MAE is 0.039 for gene expression; mean PCC is 0.967 and MAE is 0.085 for DNA methylation; Supplementary Figure S3), confirming that RFdecd’s iterative framework effectively mitigates biases from initial proportion assumptions. These findings collectively highlight RFdecd’s capacity to adaptively refine feature selection, ensuring reliable deconvolution across diverse proportion regimes and initialization scenarios.

### 3.2 Real data analysis

#### 3.2.1 Benchmarking RFdecd through seven real datasets

**FIGURE 4:**
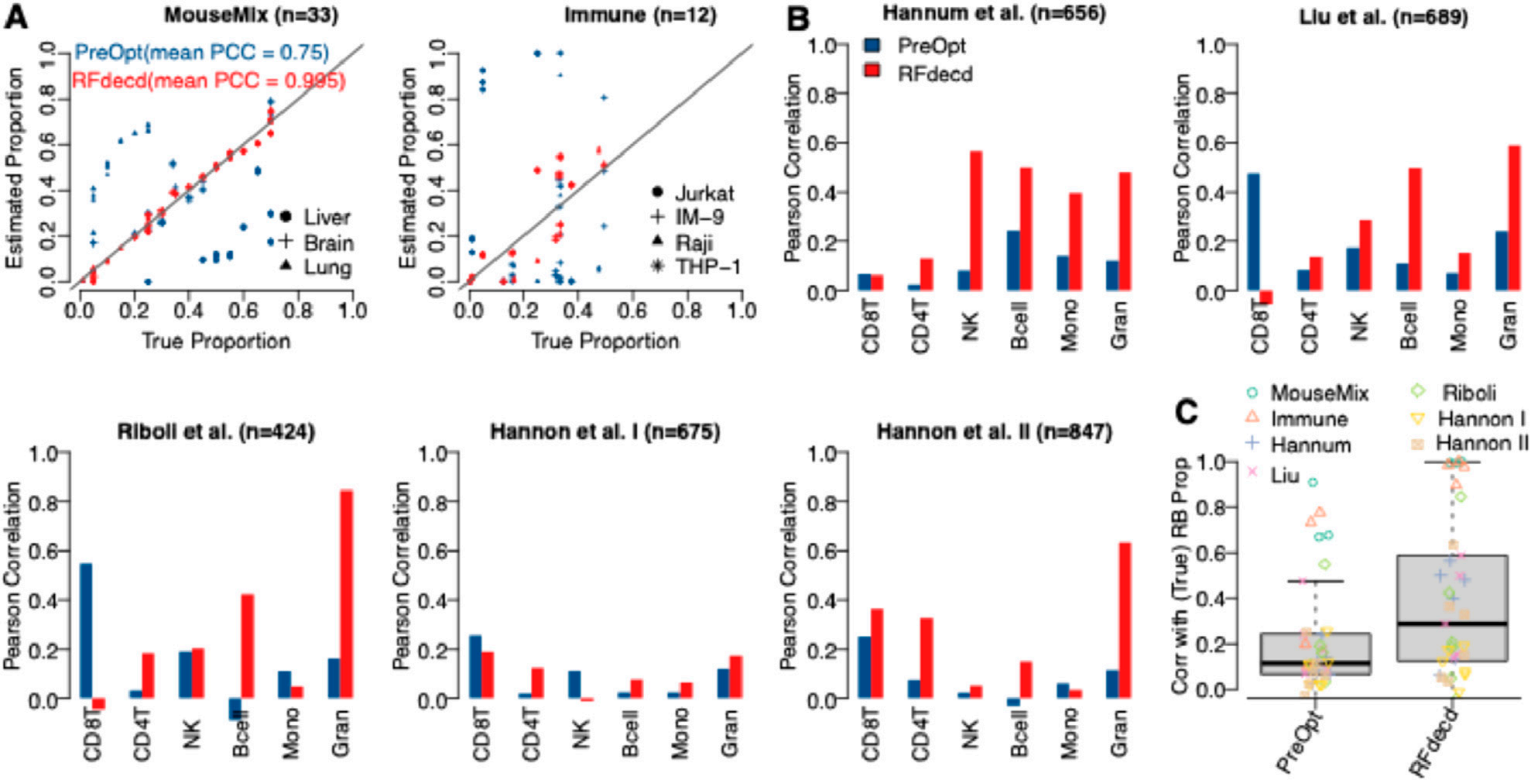
Performance assessment of RFdecd on seven real datasets. **(A)** Scatterplots depicting the estimated and true proportions of Mouse-Mix and Immune data for each cell type at the initial point (PreOpt, i.e., number of iterations = 0) and after applying RFdecd. **(B)** Bar plots illustrating Pearson correlations between reference-based resolved and estimated proportions for each cell type across the five datasets, based on the assumption of six constituent cell types (CD8T, CD4T, NK, Bcell, Mono, and Gran) in blood. **(C)** Boxplots representing Pearson correlations between the actual (or reference-based resolved) and estimated proportions for each cell type in the seven datasets. The mean Pearson correlations across cell types for Mouse-Mix and Immune data are shown in **(A)**.

Seven datasets described in Section 2.3.2 were used to evaluate the performance of RFdecd. Both Mouse-Mix and Immune data had true proportions ascertained through experiments; however, the five DNA methylation datasets did not have true proportions to provide benchmarks. To circumvent this, blood reference panels were obtained from the R package FlowSorted.Blood.450k (Jaffe and Irizarry, 2014), which furnishes methylation profiles of six cell types, namely, CD8T, CD4T, NK cell, B cell, Mono, and Gran. Subsequent to mitigating the batch effect between the mixture and reference data *via* the Combat (Johnson et al., 2007), the reference-based deconvolution method EpiDISH (Teschendorff et al., 2017) was used to derive proportion estimations. These estimated proportions were used as the reference standard for benchmarking RFdecd. Figure 4A shows the scatterplots of the estimated and true proportions of Mouse-Mix and Immune data for each cell type at the initial point (“PreOpt,” i.e., number of iterations = 0) and after applying RFdecd, and the mean Pearson correlations across cell types are shown. Improvements in proportion estimation were significant for both datasets. After applying RFdecd, mean correlations (mean PCC) increase from 0.75 to 0.995 for Mouse-Mix and from 0.451 to 0.963 for Immune data. Figure 4B shows bar plots of the Pearson correlations between the reference-based solved and estimated proportions for each cell type in the five datasets. Overall, RFdecd demonstrated superior performance compared to not using the feature-selection method, as evidenced by higher correlations between the reference-based resolved and estimated proportions for each cell type across the seven datasets. Evidently, our proposed method provides a higher mean correlation than PreOpt (0.39 versus 0.21). Moreover, we found that the correlations in Figure 4B are lower than those in Figure 4A. Nevertheless, these results still demonstrate that the proposed method achieves a significant enhancement in proportion estimation. To comprehensively evaluate the robustness of RFdecd across diverse datasets, Figure 4C presents boxplots of Pearson correlations between the actual (or reference-based resolved) and estimated proportions for each cell type aggregated across all seven datasets. Clearly, our proposed method demonstrates significant improvements in the composition estimation.

#### 3.2.2 Study of the biological significance of proportion estimation in rheumatoid arthritis

**FIGURE 5:**
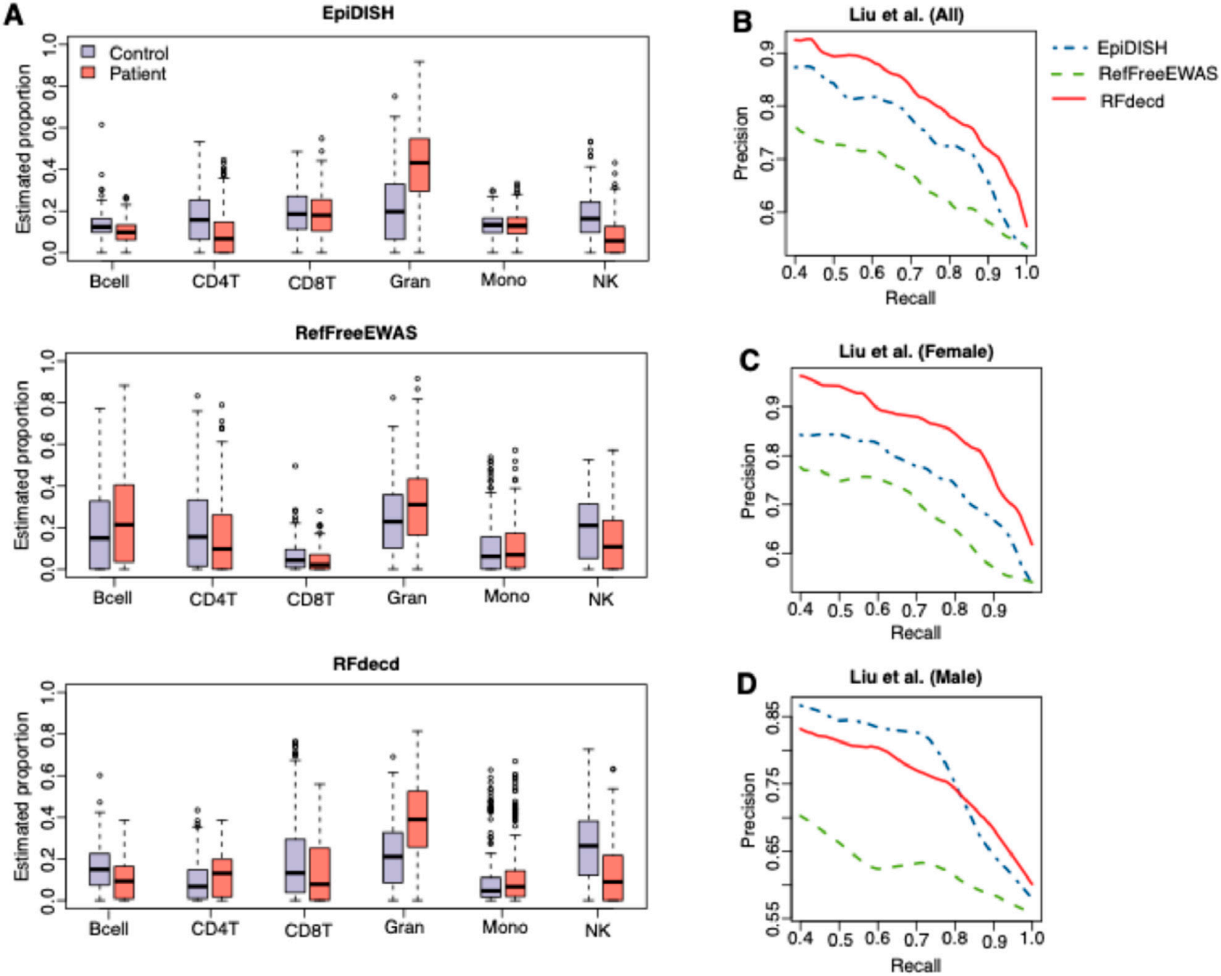
Results of the analysis of RA datasets. **(A)** Boxplots of proportion estimations of patients with RA and controls from EpiDISH, RefFreeEWAS, and RFdecd. **(B**–**D)** Precision–recall curves for predicting the disease status using estimated proportions for 689 samples **(B)**, of which 492 female samples **(C)** and 197 male samples **(D)** from the three methods. Curves were generated using the averaged results of the 10-fold cross-validation.

Finally, we examined whether the estimated proportion was biologically significant. Research has indicated that the proportions of certain blood cell types in individuals with rheumatoid arthritis (RA) deviate from those observed in healthy individuals (Hidaka et al., 1999; Kikuchi et al., 2015). Consequently, the proportion of blood cells can serve as a predictive marker for RA. Estimates of proportions that more accurately predict disease are considered to be superior. The RA dataset included 354 patients with RA and 335 healthy controls, with male and female subjects in each group (Liu et al., 2013). We used the RB method EpiDISH, along with the RF methods RefFreeEWAS and RFdecd, to decompose the 689 RA samples. The reference panel for EpiDISH was obtained from the R package FlowSorted.Blood.450k. Because the 689 samples had a disease status (control or patient), we trained a nonlinear support vector machine (SVM) with radial basis function (RBF) kernel using the estimated proportion to predict the disease status. The SVM model was trained through the “svm” function of the R package e1071, with kernel parameters optimized *via* grid search and feature vectors standardized to zero-mean unit-variance prior to model fitting. A 10-fold cross-validation was used to evaluate and compare the classification accuracies of the three methods. Figure 5A shows the estimated proportions of RA patients and controls using the three methods. Figure 5B presents the precision–recall curves for disease status prediction based on the estimated proportions of 689 samples from the three methods. It is evident that RFdecd achieved the best disease prediction performance, followed by EpiDISH and RefFreeEWAS. This result is reasonable. This is because the top 1,000 variable sites used in RefFreeEWAS contained contributions from within-cell-type variances (biological variation among samples for pure cell types), cross-cell-type variances (mean differences among pure cell types), and variation from the mixing proportions. EpiDISH is an RB method, and the reference panel provides useful information for deconvolution, resulting in a better estimation than RefFreeEWAS. When the reference panel is obtained from subjects with different phenotypes, such as age, sex, and disease status, RF can provide better proportion estimates than the RB method (Rahmani et al., 2017). In addition, RFdecd iteratively searches for cell-type-specific features and performs composition estimation, resulting in better estimation than EpiDISH. We also investigated the impact of sex on the prediction performance. Figures 5C,D show the precision–recall curves from the analysis of the RA datasets by gender. The results in Figure 5C are consistent with those in Figure 5B, indicating that RFdecd achieves better performance. Additionally, we observed greater improvements using RFdecd in female subjects than in male subjects. We believe that this could be explained by sex differences in RA etiology (Abbas et al., 2009; Affleck et al., 1999; Ahlmen et al., 2010) and sample size differences (197 male and 492 female subjects); future studies should incorporate multi-covariate analyses to disentangle the interplay of sex, age, and other clinical variables. Overall, RFdecd provides a favorable and robust performance for improving proportion estimations and disease predictions.

## 4 Discussion

In this study, we systematically evaluated five feature-selection options for reference-free deconvolution and presented an optimal feature selection-based reference-free deconvolution method, RFdecd. Our proposal iteratively searches for cell-type-specific features by integrating cross-cell-type differential analysis between one cell type and the other cell types, as well as between two cell types and the other cell types, and performs composition estimation. RFdecd does not require any prior knowledge of the cell types or their proportions; therefore, it is purely data driven. Moreover, they are not limited to specific RF deconvolution methods. Currently, most existing RF methods can be incorporated into this procedure. The application of deconf and RefFreeEWAS demonstrated this flexibility.

The proposed method is primarily aimed at microarray data of gene expression and DNA methylation. However, the idea and principle of this method can also be applied to other data types, such as RNA-seq data. A simulation study based on a real RNA-seq dataset has shown that differential analysis between one cell type and other cell types (i.e., SvC) can accurately identify cell-type-specific features (Li et al., 2019). Our current study demonstrates through comprehensive simulation analyses and real-data benchmark tests that RFdecd outperforms SvC in both cases, highlighting its stronger ability to resolve cell-type-specific features through iterative optimization. Our future work will explicitly validate these strategies in RNA-seq data.

Our selection of 1,000 features was motivated by balancing computational efficiency and biological signal preservation, which is consistent with prior genomic studies (Li and Wu, 2019). This heuristic threshold, analogous to conventional statistical cutoffs (p < 0.05), was further validated through 20 Monte Carlo simulations of 100-sample gene expression analyses (Supplementary Figure S4). We evaluated algorithm performance across varying feature numbers (500, 1,000, 1,500, up to 5,000). At 500 features, the mean correlation coefficient was 0.87, which significantly improved to 0.96 with 1,000 features. Although results for 1,500 to 4,000 features were comparable to those of 1,000 features, computational time increased by 40% (7.8 min for 1,000 features vs. 31.2 min for 4,000 features). Notably, when exceeding 4,000 features (e.g., 4,500–5,000 genes), correlation coefficients decreased. This supports 1,000 features as an optimal trade-off for balancing accuracy and efficiency in our framework. It is worth mentioning that in practical problems, this number should be case specific, and for different species, it may not hold universally.

Importantly, as discussed in recently published studies (Feng et al., 2018; Wang et al., 2019), good features for deconvolution are those with low within-cell-type variation and high cross-cell-type variation. If we select features solely based on the variance of raw observations, features with high within-cell-type variances could also be included, which would have a negative impact on the RF deconvolution in a later step, resulting in the poor performance of RefFreeEWAS. Thus, RFdecd’s iterative differential analysis framework inherently enforces this dual criterion, dynamically selecting features that maximize the biological signal while minimizing noise propagation.

Despite its merits, RFdecd has the following limitations. First, RF methods must estimate a large number of unknown parameters. Therefore, a large sample size is required to obtain accurate estimates of the cell composition. This hinders the application of RF deconvolution to small-scale studies. To evaluate this limitation, we tested RFdecd’s performance under reduced sample conditions using 30 simulated samples. As shown in Supplementary Figure S5 (parts A and B), both gene expression and DNA methylation data revealed significant improvements in mean Pearson correlations between estimated and true cell proportions across iterations for all six methods compared. Specifically, RFdecd outperformed alternatives like DvC and SvC, with mean correlations increasing from 0.85 (30 samples and 30 iterations) to 0.95 (30 samples and 100 iterations) for gene expression data (Part C), highlighting how iterative refinement mitigates sample scarcity by enhancing feature selection. Parallel analysis with 10 simulated samples demonstrated comparable trends, where 100 iterations maintained robust performance despite limited sample size, achieving a correlation of 0.93 versus 0.95 with 30 samples (Supplementary Figure S6). So, for studies with ≤50 samples, we recommend 100 iterations as a default to balance accuracy and computational efficiency. For datasets with smaller sample sizes, for example, those with fewer than 20 samples, especially those obtained from model animals (Li et al., 2020), provided a promising solution for gene expression. Second, a common challenge in applying RF methods is determining an appropriate number of cell types. For tissues that have been well studied, such as the blood and brain, prior knowledge of cell types can be easily obtained (Montano et al., 2013; Reinius et al., 2012). When there is no prior information about the number of cell types, many RF methods recommend selecting them based on model selection criteria, such as comparing the estimation error and approximation error (Lutsik et al., 2017), AIC, and BIC (Zhang et al., 2021). However, for tumor tissues, this problem is much more complicated because every two cells in the tumor tissue may be different. Under similar thresholds, cells in tumor tissues can be classified into different groups. Therefore, we propose combining model selection criteria with biological knowledge to determine the number of cell types in complex tissues. Third, assigning cell-type labels to the estimated anonymous cell types is difficult in real RF method applications. However, Rahmani et al. (2018) developed a promising Bayesian model that incorporated prior cell composition knowledge in deconvolution to solve this problem. However, prior knowledge exists only for a small number of well-studied tissues, which limits its application to real data. We provided a data-driven geometric approach to address this issue in the study of DNA methylation data (Zhang et al., 2021), which can be easily applied to gene expression data.

## Data Availability

The original contributions presented in the study are included in the article/Supplementary material; further inquiries can be directed to the corresponding author.
