# Supplementary material for: Reference-free deconvolution of complex samples based on cross-cell-type differential analysis: Systematic evaluations with various feature selection options
Source: Front Genet. 2025 May 30;16:1570781. doi: 10.3389/fgene.2025.1570781 (PMC12162504; doi:10.3389/fgene.2025.1570781)
Supplement: Supplementary file 1 [file DataSheet1.pdf]

Supplementary Material

1 Supplementary Figures and Tables

1.1 Supplementary Table

The proposed method operates through three sequential phases (Algorithm 1):

1. Initialization: Selects the top 1000 features (M_0) with the highest coefficient of variation (CV) from the raw data matrix Y, performs reference-free (RF) deconvolution on the reduced matrix Y_{M_0} to estimate initial cell-type profile matrix W_1 and proportion matrix H_1, and calculates the reconstructed error.

2. Iterative optimization: Cyclically updates the feature list M_i using six feature selection options (VAR, CV, SvC, DvC, PwD and RFdecd), re-estimates W_{i+1} and H_{i+1} through RF deconvolution on Y_{M_i}, and updates RMSE[i+1] across iterations (1$\leq i \leq totalIter$).

3. Termination: Identifies the optimal proportion matrix H_{id} corresponding to the iteration with minimal RMSE.

Algorithm 1: Iterative feature optimization in RF deconvolution

Input: Raw data matrix $\boldsymbol{Y}$

Output: Cell-type proportion matrix $\boldsymbol{H_{id}}$

Notations:

$\boldsymbol{K}$: Number of cell types;

totalIter**:** Number of iterations;

$\boldsymbol{m}$: Number of features;

$\boldsymbol{n}$: Number of samples;

$\boldsymbol{M_i}$**:** Cell-type specific feature list at iteration i;

$\boldsymbol{Y_{M_i}}$**:** Submatrix of Y with features in M_i;

$\boldsymbol{W_i}, \boldsymbol{H_i}$**:** Cell-type profile and proportion matrix at iteration i;

$\boldsymbol{RMSE[i]} = \sqrt{\sum(\boldsymbol{Y} - \boldsymbol{W_i H_i})^2/\boldsymbol{mn}}$**:** Reconstruction error;

Step1: Initialization

1a. Feature Selection:

Select top 1000 features with the largest coefficient of variation (CV) on Y as M_0;

1b. Initial deconvolution

Conduct RF deconvolution on Y_{M_0} with K cell types to estimate initial cell proportions H_1, followed by applying the csfit function from csSAM package to derive cell-type profiles (W_1);

1c. Error calculation:

Compute $RMSE[1] = \sqrt{\sum(Y - W_1H_1)^2/mn}$;

Step2: Iterative optimization

For $i = 1$ to *totalIter*:

2a. Feature update:

Input: H_i, Y;

Feature selection options:

VAR/CV: Select top 1000 features from W_i based on VAR or CV;

SvC, DvC, PwD: Perform cross-cell type differential analysis (Eq. 3-5) and retain top $\left\lceil\frac{1000}{K} * 1.2\right\rceil$ features per cell type;

RFdecd: Hybrid selection combining SvC and DvC;

Output: Updated feature list M_i;

2b. Deconvolution:

Apply RF deconvolution on Y_{M_i} with K cell types to estimate W_{i+1} and H_{i+1};

2c. Error update:

Compute $RMSE[i + 1]$=$\sqrt{\sum(Y - W_{i+1}H_{i+1})^2/mn}$;

Step3: Termination

3a. Optimal Solution:

Let $id = \text{argmin}_\text{i} RMSE[i]$;

Return H_{id}.

1.2 Supplementary Figures

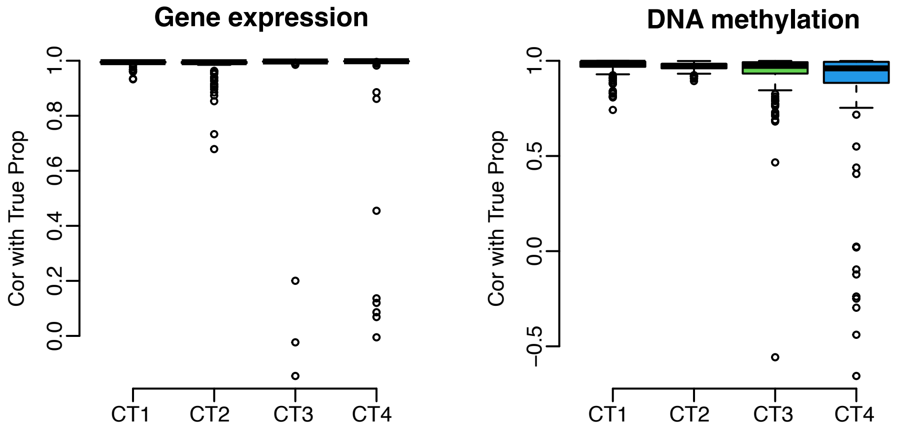

Supplementary Figure 1. Validation of 30 iterations for stable proportion estimation. Performance evaluation on synthetic mixtures generated from the gene expression dataset (GSE19830) and DNA methylation dataset (GSE35069). Boxplots depict correlations between estimated and true cell-type proportions for features selected at the 30th iterations. Results are aggregated from 100 Monte Carlo simulations with a sample size of 100 per simulation.

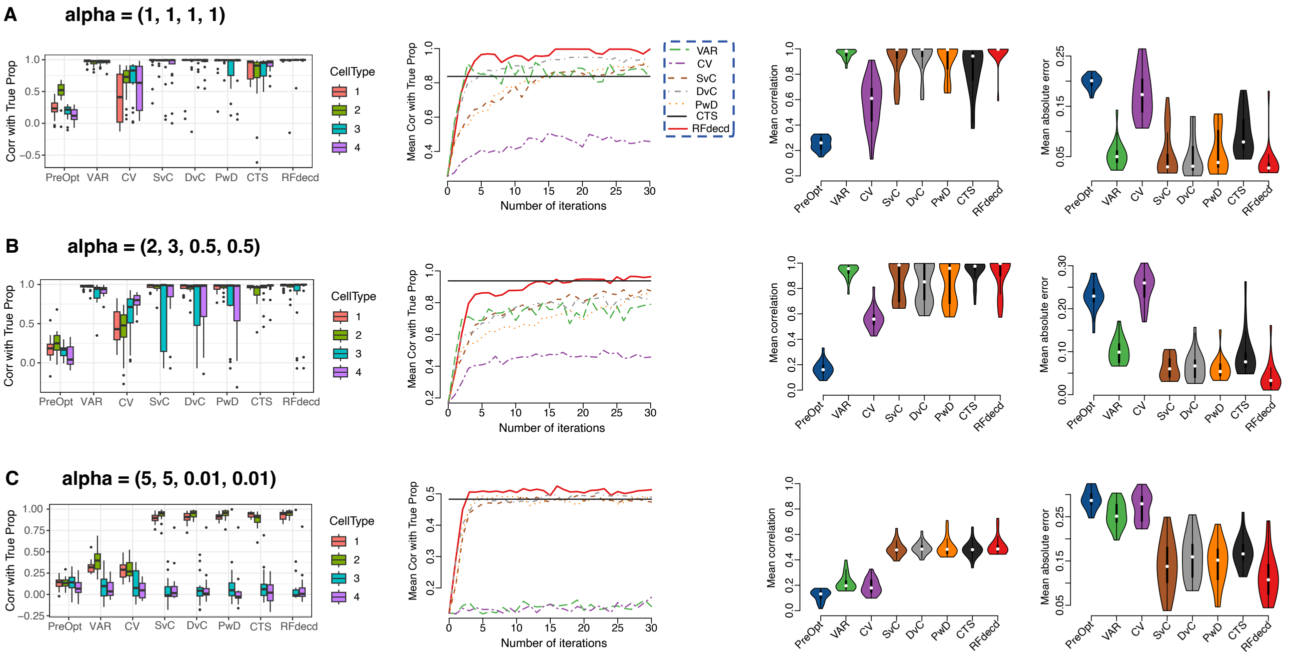

Supplementary Figure 2. Performance evaluation of eight deconvolution algorithms on synthetic mixtures generated from gene expression dataset (GSE19830). The analysis was conducted under three distinct Dirichlet parameter configurations to simulate varying cell-type proportion scenarios: (A) a uniform distribution (1, 1, 1, 1) modeling balanced cell-type proportions, (B) a moderately skewed distribution (2, 3, 0.5, 0.5) reflecting intermediate variability in cell-type abundance, and (C) an extreme distribution (5, 5, 0.01, 0.01) including near-zero proportions for two cell types. For each scenario, the left panel display boxplots of Pearson correlations between estimated and true proportions for four cell types. The second panel illustrates the mean Pearson correlations across four cell types by number of iterations. The third and fourth panels present the mean Pearson correlation and mean absolute error (MAE), respectively, between estimated and true proportions across the four cell types. The CTS method utilized 1000 real cell type-specific (CTS) features derived from pure cell type profiles for direct proportion estimation via RF deconvolution, while the PreOpt method employed top 1000 CV-selected features for RF-based proportion estimation. Results for the six iterative methods represent the lowest RMSE across 30 iterations. All metrics are aggregated from 20 Monte Carlo simulations with a sample size of 100 per simulation.

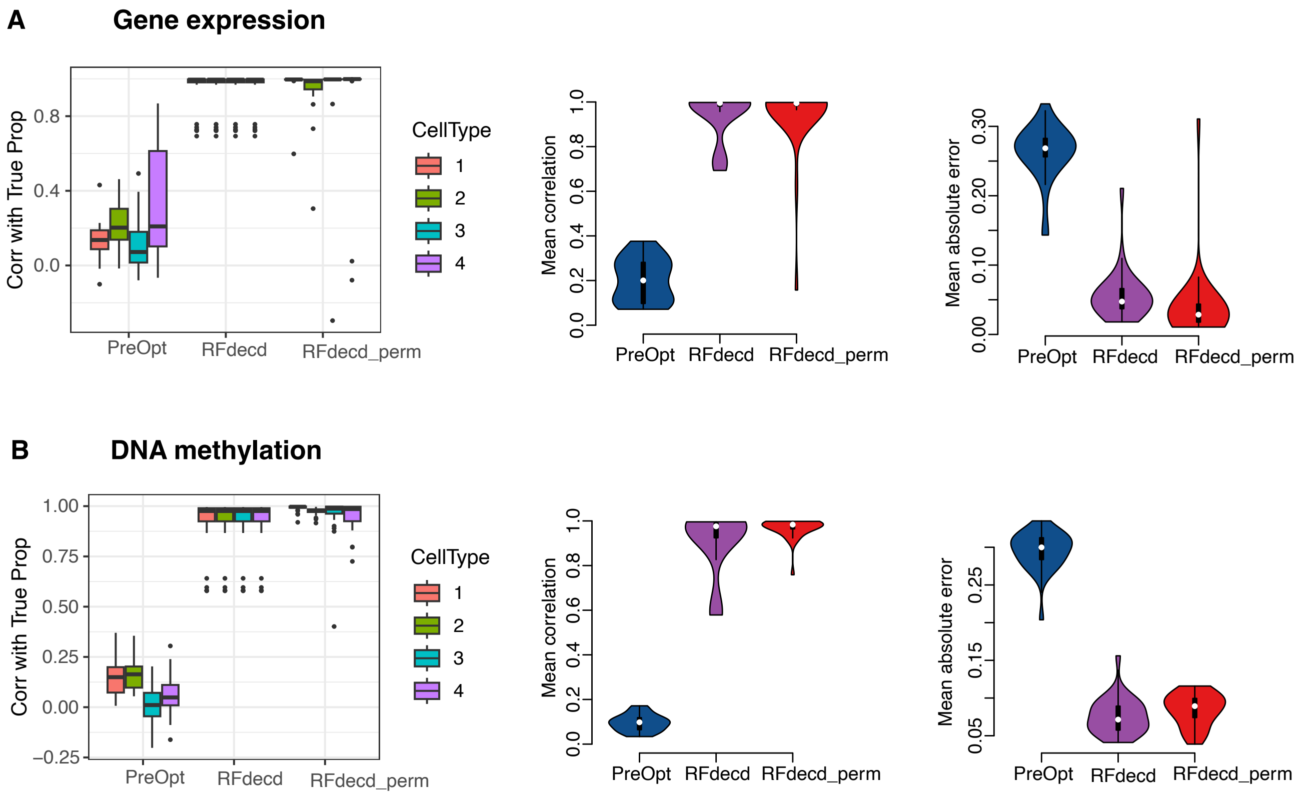

Supplementary Figure 3. Robustness assessment of the iterative deconvolution framework against perturbations in the initial proportion matrix. Synthetic mixtures were generated using (A) the gene expression dataset (GSE19830) and (B) the DNA methylation dataset (GSE35069) to evaluate the impact of randomized initialization ("RFdecd-perm"). Left panels: boxplots of mean Pearson correlations between estimated and true cell-type proportions across four cell types over 30 iterations. Middle and right panels: boxplots comparing mean Pearson correlations (middle) and mean absolute errors (right) for the three methods, including the baseline "PreOpt" (initial proportion estimation using top 1000 CV-selected features without iteration). Results for RFdecd and RFdecd-perm reflect the lowest RMSE across 30 iterations. All metrics are aggregated from 20 Monte Carlo simulations with 100 samples per simulation.

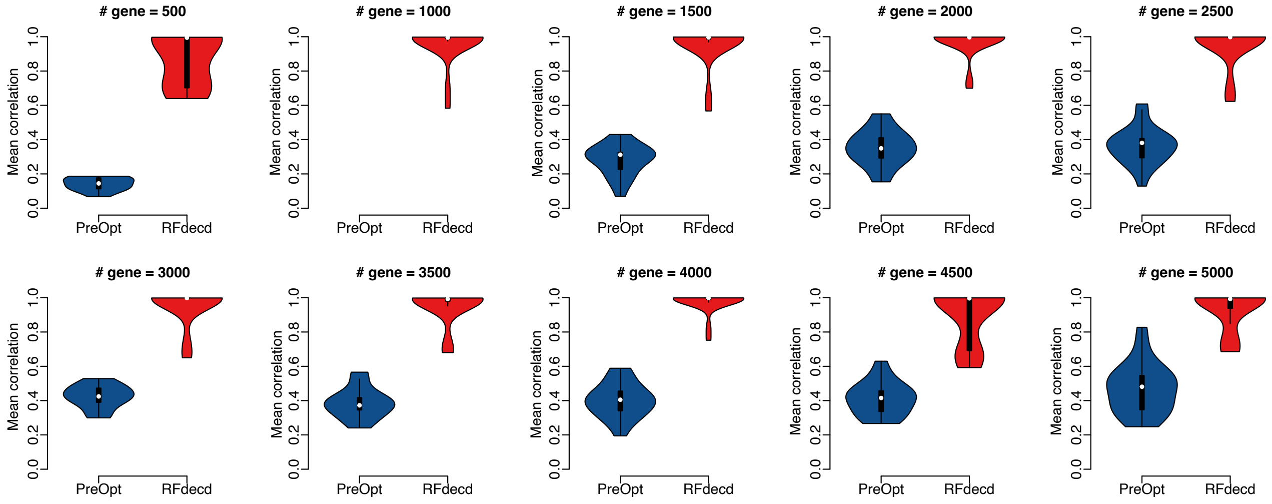

Supplementary Figure 4. Performance evaluation of feature quantity impact on deconvolution accuracy in synthetic mixtures generated from gene expression dataset (GSE19830). Panel sequentially represent analyses using 500, 1000, 1500, up to 5000 features. Each panel display boxplots of the mean Pearson correlations between estimated and true cell-type proportions across four cell types over 30 iterations. The presented results are summarized over 20 Monte Carlo experiments with 100 samples per simulation.

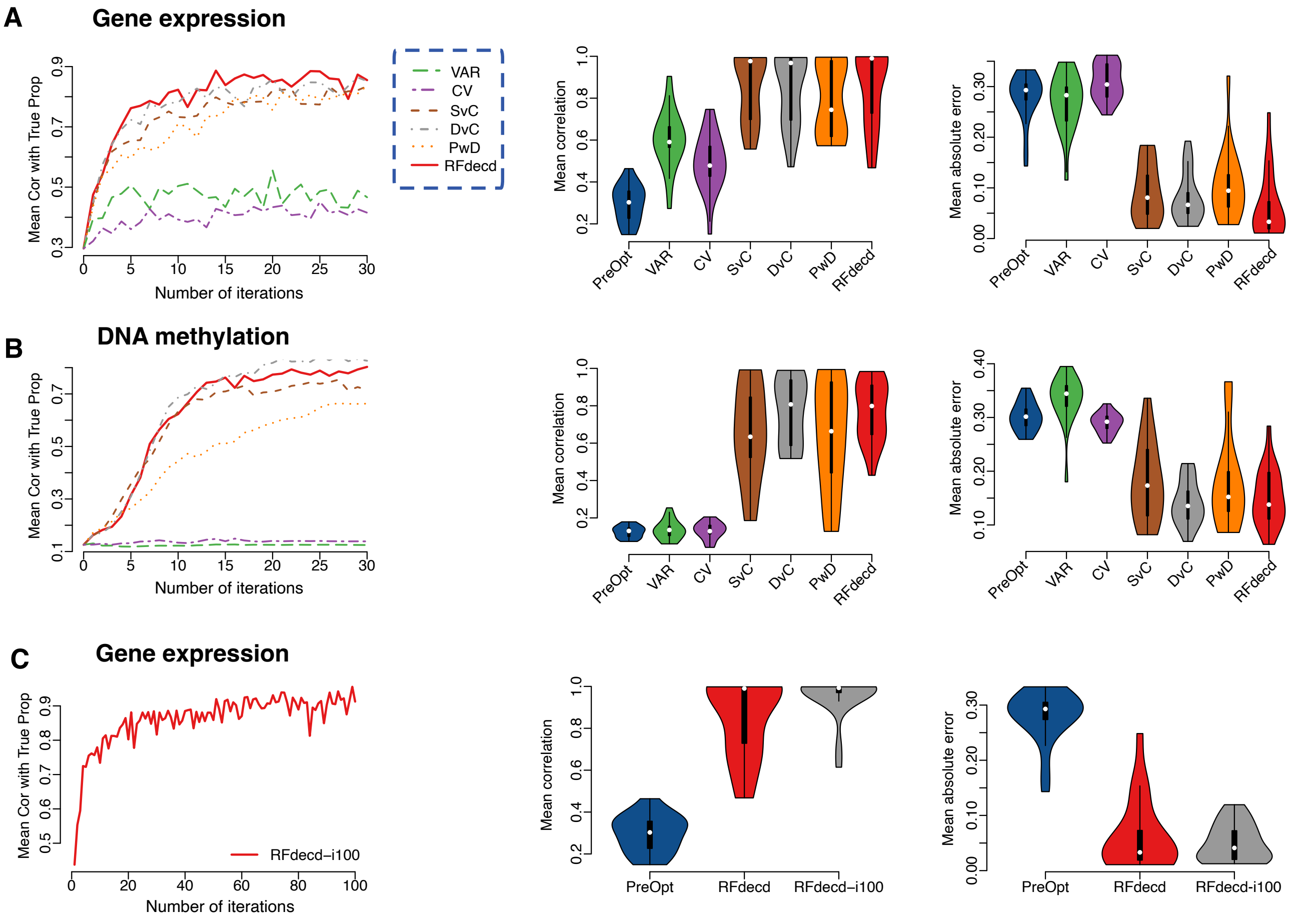

Supplementary Figure 5. Performance evaluation of deconvolution accuracy when the sample size in synthetic mixtures is 30. Synthetic mixtures were generated using (A) the gene expression dataset (GSE19830, 30 iterations) and (B) the DNA methylation dataset (GSE35069, 30 iterations). Left: mean Pearson correlations across four cell types by number of iterations. Middle and right: boxplots comparing seven deconvolution methods, including the baseline "PreOpt" (initial proportion estimation using top 1000 CV-selected features without iteration) for mean Pearson correlations (middle) and mean absolute errors (MAE, right). Results for comparative methods reflect the lowest RMSE across 30 iterations. (C) Synthetic mixtures generated from gene expression dataset (GSE19830) with 100 iterations. Left: mean Pearson correlations across four cell types over 100 iterations for RFdecd-i100 (100 iterations). Boxplots comparing three methods (PreOpt: baseline method using top 1000 CV-based features; RFdecd: standard implementation with 30 iterations; enhanced implementation with 100 iterations) for mean Pearson correlation (middle) and MAE (right). All metrics are aggregated from 20 Monte Carlo simulations with 100 samples per simulation.

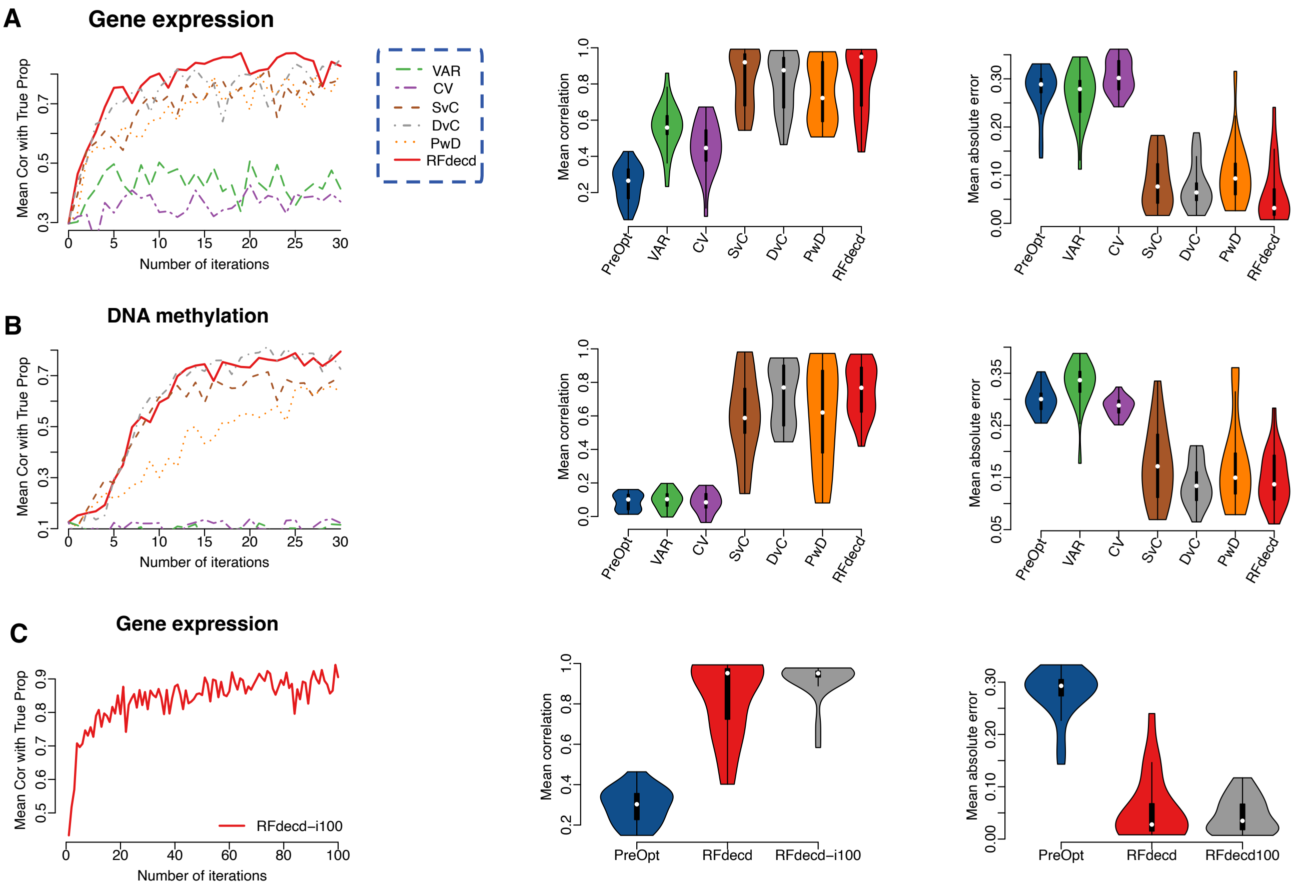

Supplementary Figure 6. Performance evaluation of deconvolution accuracy when the sample size in synthetic mixtures is 10. Synthetic mixtures were generated using (A) the gene expression dataset (GSE19830, 30 iterations) and (B) the DNA methylation dataset (GSE35069, 30 iterations). Left: mean Pearson correlations across four cell types by number of iterations. Middle and right: boxplots comparing seven deconvolution methods, including the baseline "PreOpt" (initial proportion estimation using top 1000 CV-selected features without iteration) for mean Pearson correlations (middle) and mean absolute errors (MAE, right). Results for comparative methods reflect the lowest RMSE across 30 iterations. (C) Synthetic mixtures generated from gene expression dataset (GSE19830) with 100 iterations. Left: mean Pearson correlations across four cell types over 100 iterations for RFdecd-i100 (100 iterations). Boxplots comparing three methods (PreOpt: baseline method using top 1000 CV-based features; RFdecd: standard implementation with 30 iterations; enhanced implementation with 100 iterations) for mean Pearson correlation (middle) and MAE (right). All metrics are aggregated from 20 Monte Carlo simulations with 100 samples per simulation.
